# Retrospective analysis of SARS-CoV-2 omicron invasion over delta in French regions in 2021–22: a status-based multi-variant model

**DOI:** 10.1186/s12879-022-07821-5

**Published:** 2022-11-03

**Authors:** Thomas Haschka, Elisabeta Vergu, Benjamin Roche, Chiara Poletto, Lulla Opatowski

**Affiliations:** 1 Epidemiology and Modelling of Bacterial Escape to Antimicrobials, Institut Pasteur, 25-28, rue du Docteur Roux, 75015 Paris, France; 2 CESP, Anti-infective evasion and pharmacoepidemiology research team, U1018, INSERM Université Paris-Saclay, UVSQ, 2, Avenue de la Source de la Bièvre, 78180 Montigny-Le-Bretonneux, France; 3 Université Paris-Saclay, INRAE, MaIAGE, Domaine de Vilvert, 78350 Jouy-en-Josas, France; 4 MIVEGEC, Université Montpellier, IRD, CNRS, 911, avenue Agropolis, 34394 Montpellier, France; 5 INSERM, Institut Pierre Louis d’Epidémiologie et de Santé Publique, Sorbonne Université, 75012 Paris, France

**Keywords:** Multi-variant model, SARS-CoV-2, Omicron, Transmissibility

## Abstract

**Background:**

SARS-CoV-2 is a rapidly spreading disease affecting human life and the economy on a global scale. The disease has caused so far more then 5.5 million deaths. The omicron outbreak that emerged in Botswana in the south of Africa spread around the globe at further increased rates, and caused unprecedented SARS-CoV-2 infection incidences in several countries. At the start of December 2021 the first omicron cases were reported in France.

**Methods:**

In this paper we investigate the spreading potential of this novel variant relatively to the delta variant that was also in circulation in France at that time. Using a dynamic multi-variant model accounting for cross-immunity through a status-based approach, we analyze screening data reported by *Santé Publique France* over 13 metropolitan French regions between 1st of December 2021 and the 30th of January 2022. During the investigated period, the delta variant was replaced by omicron in all metropolitan regions in approximately three weeks. The analysis conducted retrospectively allows us to consider the whole replacement time window and compare regions with different times of omicron introduction and baseline levels of variants’ transmission potential. As large uncertainties regarding cross-immunity among variants persist, uncertainty analyses were carried out to assess its impact on our estimations.

**Results:**

Assuming that 80% of the population was immunized against delta, a cross delta/omicron cross-immunity of 25% and an omicron generation time of 3.5 days, the relative strength of omicron to delta, expressed as the ratio of their respective reproduction rates, $\frac{\hat{R}_{\textrm{omicron}}}{\hat{R}_{\textrm{delta}}}$, was found to range between 1.51 and 1.86 across regions. Uncertainty analysis on epidemiological parameters led to $\frac{\hat{R}_{\textrm{omicron}}}{\hat{R}_{\textrm{delta}}}$ ranging from 1.57 to 2.34 on average over the metropolitan French regions, weighted by population size.

**Conclusions:**

Upon introduction, omicron spread rapidly through the French territory and showed a high fitness relative to delta. We documented considerable geographical heterogeneities on the spreading dynamics. The historical reconstruction of variant emergence dynamics provide valuable ground knowledge to face future variant emergence events.

**Supplementary Information:**

The online version contains supplementary material available at 10.1186/s12879-022-07821-5.

## Background

The SARS-CoV-2 pandemic first emerged in China in December 2019 and subsequently spread all over the world. Despite unprecedented control measures and availability of several vaccines, the virus persisted in populations and evolved into different lineages. These SARS-CoV2 mutations have been classified into different variants that have caused further isolated or overlapping epidemic waves in many countries [1]. In particular, variants with increased transmissibility, increased severity or immune escape were observed, and defined to be variants of concern (VOC). These VOCs were further named after the letters of the Greek alphabet.

The omicron SARS-CoV-2 VOC, first detected in Botswana in the south of Africa [2] in November 2021, spread rapidly around the world [3, 4]. The mutations observed on this variant are expressing a different form of the Spike protein [5], seemingly causing immune escape [6] and higher infection rates [7–10].

The detection of the omicron variant in France was noticed at the start of December 2021 [11]. Early assessments of its dynamics pointed to a rapid growth and hence, a substantial spreading advantage over the delta variant, the circulating variant at that time in France [12, 13]. Therefore, omicron has been attributed the potential to cause a large-scale epidemic wave [12–14]. The rate of daily detected cases, indeed, underwent unprecedented growth and over 300,000 detected cases per day were registered in the first half of January for this country consisting of a population of almost 67 million inhabitants [15]. Here, we retrospectively analysed the dynamics of the emergence of the omicron variant and the replacement of the delta variant across all French regions.All the data that we have at our disposal from *Sante Publique France*, which is further detailed in section 2.1, highlights only a negligible proportion of observed omicron samples at the beginning of December, and an almost 100% occurrence of omicron samples at the end of January for all French metropolitan regions. Therefore, we focused our study on a time-frame between December 2021 and January 2022. According to https://outbreak.info [16–18] and [19, 20] lineages besides BA.2 did not yet emerge in significant rates during that period. Lineage BA.2 itself did not account for more then 10% of samples by the end of January 2022 and we focused therefore on an omicron invasion on French territory that is BA.1 based.

In order to perform this study we developed a status-based multi-variant compartmental model which was built upon [21]. This model allows us to simulate the coevolution of multiple variants independently and link them with an interaction matrix, accounting for cross immunity between the different variants. Hence, the model is perfectly adapted for the situation where one variant is replaced by another. Fitting this model to the data observed in France during the winter of 2021/2022 allows us to quantify the relative advantage of the omicron variant over the delta variant and its spatial heterogeneity on the replacement dynamics, by accounting for uncertainty in different factors, such as the generation time of a specific variant.

## Methods

### Data acquisition and preprocessing

PCR-confirmed cases associated with SARS-CoV-2 mutations were obtained from *Santé Publique France*^1^ for the 13 metropolitan French regions. The data includes the number of tests that underwent the screening for a selection of mutations in their amino acid sequence. Different mutations were monitored for their impact on viral functioning and because they were recognised as indicators of different VOCs. In particular, the E484K mutation is commonly used as an indicator of a beta or gamma variant and the L452R as an indicator of the delta variant. The absence of these two mutations is characteristic of the alpha, omicron or other lineages, e.g. B.1.640. Periodic whole genome sequencing surveys showed that B.1.640 was circulating at a low level in France at the time of omicron introduction to be rapidly replaced by omicron around mid December [12, 13]. The omicron lineage BA.5 could exert the L452R mutation but were according to [16–18] not, or only in negligible quantities [19, 20], present in metropolitan France in the investigated period. In late November 2021, a dedicated surveillance protocol was established in France targeting a set of mutations specific to the omicron variant. The initial set of mutations was soon updated to become in late December: deletion of site 69/70 and/or substitutions K417N and/or S371L-S373P and/or Q493R [22]. The protocol was initially adopted by certain laboratories to become progressively more widespread throughout December, early January.

Available records were used to describe the co-circulation of omicron, delta and beta/gamma. Records with L452R mutation were interpreted as delta variant. E484K mutations were taken as indicator of beta/gamma variant. These were counted in negligible fractions but were kept for completeness. For omicron the two alternative options for data interpretations were subject to different potential biases. The use of the absence of L452R and E484K mutations as a proxy for omicron was biased around the onset of omicron invasion due to the co-circulation with other lineages. Given that omicron became dominant among the samples without L452R mutation at the very beginning of its invasion, as explained above, this biases had likely a limited effect on the whole replacement curve. On the other hand, the use of the omicron-specific set of mutations was likely biased during the period from the end of November and beginning of January when such an indicator was adjusted and gradually adopted throughout the French territory. Given our interest on the entire replacement period between the beginning of December and the end of January, we assumed in the baseline analysis omicron to be described by the absence of L452R and E484K mutations. Still, we considered the alternative indicator in the sensitivity analysis.

**Fig. 1:**
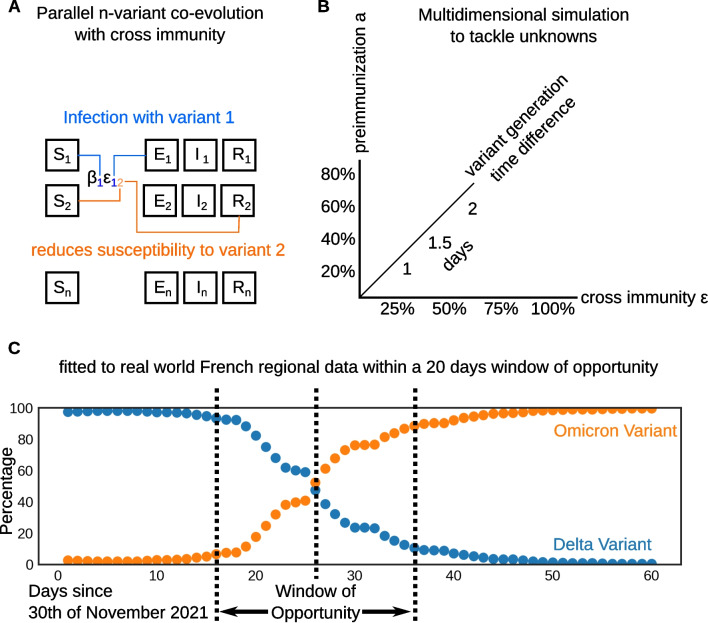
**A** A basic overview of the model. **B** The model paramterization possibilities. **C** As an illustration, percentages of delta and omicron obtained from *Santé Publique France* for the Ile-de-France region with the 20 day window of opportunity around the inflection point as it has been chosen for this modelling study

Visual inspection of the time series between 1st December 2021 and 31th January 2022 reveals that the invasion of omicron, more precisely the lineage BA.1, occurs approximately in three weeks in all the regions of metropolitan France. As such we defined for each region a window of opportunity of 20 days for the analysis. The onset of this window is defined by its midpoint, the 10th day, where omicron shall supplant the delta variant in absolute numbers, meaning that the omicron variant exceeds 50% of reported samples at this midpoint. This approach is outlined in Fig. 1 which represents the data of a typical French region and its selected 20 day window of opportunity (Fig. 1C). The dataset provided by *Santé Publique France* is smoothed over a 7-day sliding window.

### Multi-variant transmission model

We modelled the co-circulation of three variants. Inspired by [21], we proposed a status-based multi-variant compartmental model allowing us to evaluate the delta, omicron and residual beta/gamma variants simultaneously, which interact with each other using a cross-immunity term. A schematic overview of the model is shown in Fig. 1A. As outlined in this figure the main advantage of this model is that allows us to simulate multiple variants simultaneously taking the immunity acquired, due to the infection by one variant against an other variant into account. This property is modeled using a cross-immunity matrix $\epsilon _{ik}$. The authors of [21] have proposed a similar parsimonious process to model a large number of competing strains, with application to the influenza dynamics. We largely adapted this model in our study for the purpose of describing the dynamics of competing SARS-CoV-2 variants. The model is defined by the ordinary differential eqs. (1–4), where state variables stand for proportions of different compartments in the population from the viewpoint of each variant *i*:

1 $$\begin{aligned} dS_{i}= \,& {} \left( \eta _{i}R_{i}-S_{i}\sum _{k=1}^n \epsilon _{ik}\beta _{k}I_{k} \right) dt, \end{aligned}$$

2 $$\begin{aligned} dE_{i}= \,& {} \left( \beta _{i}S_{i}I_{i}-\delta _i E_{i}\right) dt, \end{aligned}$$

3 $$\begin{aligned} dI_{i}= \,& {} (\delta _i E_{i} - \gamma _i I_{i})dt, \end{aligned}$$

4 $$\begin{aligned} R_{i}= \,& {} 1-S_{i}-E_{i}-I_{i}. \end{aligned}$$

$S_i$ represents the population susceptible, $E_i$ the incubating non infectious population and $I_i$ the population of infectious individuals. Compartment $R_i$ models an immunized population that either underwent infection and recovered from the disease or has been vaccinated. $\beta _i$ represents the transmission rate, $\eta _i$ is the immunity waning rate, $\delta _i$ the rate at which exposed individuals become infectious, or the inverse of the mean sojourn time in *E* compartment, and $\gamma _i$ the recovery rate or the inverse of the infectiousness duration. Variant interaction is modelled by a cross-immunity matrix, where element $\epsilon _{ik}$ describes the acquired protection to an acquisition of variant *i* conferred by an infection with variant *k*. For a given reproduction rate at time *t*, $\hat{R}_k(t)$, the transmission rate $\beta _{k}$ can be obtained from:

5 $$\begin{aligned} \beta _{k} = \frac{\hat{R}_k(t=0)\gamma _k}{1-a_{k}}, \end{aligned}$$

where $a_k$ represents the immunization level in the population against variant *k* at the beginning of the study period. In further detail, in eq. (1) the $S_{i}\sum _{k=1}^n \epsilon _{ik}\beta _{k}I_{k}$ term models the cross-immunity and, at the same time, the exit from the susceptible compartment of newly infected individuals. It is therefore the most significant part of this model. Aforementioned $\beta _{k}$ as outlined in eq. (1), modulates the strength of the infection, while the $\epsilon _{ik}$ matrix makes sure that variants *i* are also affected by a pull resulting from infected individuals $I_{k}$, and takes, as such, care about the cross-immunity. Further equations of the model (2,3,4) follow standard SEIR modelling procedures, with the main difference being that index *i* accounts for multiple variants in coevolution. In the case of the omicron variant, the immunized fraction $a_{\textrm{omicron}}$ is obtained by multiplying the fraction of population immune against the delta variant $a_{\textrm{delta}}$ with the cross-immunity between omicron and delta:

6 $$\begin{aligned} a_{\textrm{omicron}} = a_{\textrm{delta}}\epsilon _{\mathrm {[delta,omicron]}}. \nonumber \end{aligned}$$

We assume that $\hat{R}_k(t)$ is constant over the investigated period, and we define the relative epidemic fitness of variant *i* to the delta variant as the ratio of reproduction rates: $\frac{\hat{R}_i}{\hat{R}_{\textrm{delta}}}$.

Parameter values were either (i) based on literature values in the case of $\delta _i$ and $\gamma _i$, or (ii) hypothesised for $a_k$, $\eta _{i}$ and $\epsilon _{ik}$, with different values tested for robustness purposes for both (i) and (ii) (see Sect. 2.4), or (iii) estimated from data for $\beta _i$ [related to $\hat{R}_k$, equation (5)]. In the baseline scenario, the mean generation time, which is expressed in our model for a variant *i* as $1/\delta _i + 1/\gamma _i$, was assumed equal to 5 days for delta, 3.5 days for omicron [23] and 8 days for the other variants (beta/gamma) [24, 25]. The corresponding durations ($1/\delta _i$ and $1/\gamma _i$) in $E_i$ and $I_i$ compartments were assumed equal to (2,3) days, (1.4, 2.1) days and (5,3) days for delta, omicron and beta/gamma variants, respectively. A further in detail overview of all rate constants evaluated is outlined in Table 1.

**Table 1:** Values used in the sensitivity analysis for mean generation time and duration in *E* and *I* compartments for each variant, from [23–25]. All values are given in days

Description	Delta	Omicron
Mean generation time	5	3
Mean duration in *E* $1/\delta$	2	1.2
Mean infectiousness duration $1/\gamma$	3	1.8
Mean generation time	5	3.5
Mean duration in *E* $1/\delta$	2	1.4
Mean infectiousness duration $1/\gamma$	3	2.1
Mean generation time	5	4
Mean duration in *E* $1/\delta$	2	1.6
Mean infectiousness duration $1/\gamma$	3	2.4
Mean generation time	5	6
Mean duration in *E* $1/\delta$	2	2
Mean infectiousness duration $1/\gamma$	3	4
Mean immunity duration $1/\eta$	1000	1000

The model is integrated using a Runge-Kutta-Fehlberg (RKF) algorithm with variable step size [26]. Further we interpolate a third order polynomial on subsequent successions of four obtained data points. This allows us to extract a value on the continuum between the start and the end of the simulation performed with the model described herein. The source code for the model, written in C, has been made available on Github^2^.

### Fitting the model to the data and initial conditions

The model was fitted to the reported proportions of sampled variants for each French metropolitan region independently. The fit was performed on a 20 days window starting 10 days prior the inflection point where the omicron variant is present in more than half of the samples.

For all metropolitan French regions, initial conditions were obtained by collecting public estimates of SARS-CoV-2 incidence and reproduction rates, for all variants combined, for the 6th, 13th and 20th of December from *Santé Publique France*^3^. More precisely, the initial conditions were computed based on this data as follows: for each region, $t=0$ represents the first day of the 20-day study period in our simulations. Depending on the date of the initial point of the study period, we define $q(t=0)$ and $\hat{R}_{\textrm{delta}}(t=0)$ to be the linear interpolations of these collected estimates for each region. If the first day of the 20 days of the simulated window happens before the 6th of December or after the 20th of December, no interpolation is performed and the values from the respective day are taken. Visual inspection confirms that 10 days prior the inflection point, at the beginning of our window of opportunity, omicron cases were still very rare. As such, the interpolated reproduction rate $\hat{R}$ was entirely attributed to the delta variant and stayed constant through the study period.

For each region-associated time window, we used the obtained initial incidence rate $q(t=0)$ to set the initial conditions of compartments $E_i(t=0)$ and $I_i(t=0)$ as follows:

$$\begin{aligned} E_i(t=0)= & {} \left\{ \begin{array}{l} i = 1 \mathrm {(delta)}: \frac{1}{\delta }q(t=0)\frac{P_i(t=0)}{\sum _{i=1}^n P_i(t=0)} \\ i \ne 1 \mathrm {(other)}: \frac{u_i}{\delta }q(t=0)\frac{P_i(t=0)}{\sum _{i=1}^n P_i(t=0)} \end{array}, \right. \\ I_i(t=0)= & {} \left\{ \begin{array}{l} i = 1 \mathrm {(delta)} : \frac{1}{\gamma } q(t=0)\frac{P_i(t=0)}{\sum _{i=1}^n P_i(t=0)} \\ i \ne 1 \mathrm {(other)} : \frac{u_i}{\gamma } q(t=0)\frac{P_i(t=0)}{\sum _{i=1}^n P_i(t=0)} \end{array}, \right. \end{aligned}$$

where $P_i$ is the number of positive samples of variant *i* found in the data at the onset date of our study period. $u_i$ is a fit parameter which defines the initial proportion, for each variant *i* except delta variant.

Curve fitting was achieved using a standard gradient descent procedure. Parameters related to VOCs other than the delta variant, which was kept static, were optimized by minimizing the following loss function independently for each region:

7 $$\begin{aligned} L = \sum _{j=1}^n\sum _{l=1}^k\left| \frac{P_j(t_l)}{\sum _{i=1}^n P_i(t_l)}-\frac{\delta _jE_{j}(G,t_l)}{\sum _{i=1}^n\delta _i E_{i}(G,t_l)}\right| , \end{aligned}$$

for *n* variants and *k* sampled moments in time. Here $P_i$ represents the observed data, as defined above, and $\delta _j E_{j}(G,t_l)$ the simulated incidence of new infectious individuals at time point $t_l$ as described by eq. (2), where $G = \{\hat{R}_i, u_i : i \ne 1 \text{(variants } \text{ other } \text{ than } \text{ delta) }\}$. Parameters minimizing the loss function *L* (6) were estimated. Mean estimates as well as standard variations across regions for the omicron reproduction ratio were calculated. We also computed estimates weighted according to the regions population size:

8 $$\begin{aligned} \bar{R}= & {} \frac{1}{N} \sum _{\textrm{reg}} N_{\textrm{reg}}\hat{R}_{\textrm{reg}},\\ \sigma _R= & {} \sqrt{\frac{1}{N} \sum _{\textrm{reg}} N_{\textrm{reg}}(\hat{R}_{\textrm{reg}}-\bar{R})^2}, \end{aligned}$$

with *N* representing the total population of metropolitan France and $N_{\textrm{reg}}$ the population of a single region *reg*. $\hat{R}_{\textrm{reg}}$ is the best result yielded by the gradient descent for $\hat{R}_{\textrm{omicron}}$ obtained for the corresponding region.

As outlined in Fig. 1B, independent fits were performed in conjunction with parameter sweeps to overcome uncertainties as further detailed in Sect. 2.4.

### Sensitivity analysis

As uncertainty still exists regarding some model parameters such as the generation time and few information is available on others such as variant-specific immunity in the population, we performed a sensitivity/uncertainty analysis to investigate the impact of the different model parameters on our estimates and subsequent variant dynamics.

More precisely, we varied: (i) the generation time of the omicron variant setting it to equal to 3, 3.5, 4 and 6 days as shown in Table 1 which summarizes tested values, (ii) the acquired immunity in the population *a* against variants prior to omicron obtained either by infection or vaccination, with $a \in (0.2, 0.4, 0.6, 0.8)$, and (iii) the cross-immunity $\epsilon$ that this immunity confers to the omicron variant, with $\epsilon \in (0.25, 0.5, 0.75, 1.0)$.

First, the impact of varying model parameters in terms of model fitting was explored for all parameters (i)–(iii).

Second, the impact of the uncertainty in model parameters on replacement dynamics was investigated. When a new variant replaces the established variant, we can numerically quantify the relative fitness by means of $\Delta t$, i.e. the time it takes for a new variant to rise from 10% to 50% of positively tested cases in a population. This idea is illustrated in Fig. 4A, for both a less fitter new variant (orange) characterised by $\Delta t_1$ and a stronger new variant (blue) characterised by $\Delta t_2$, respectively. To get a better understanding of the relationship between our fitness estimates and parameters *a* (ii) and $\epsilon$ (iii), we analysed in the model variations of:

9 $$\begin{aligned} \Delta t_{10\%-50\%}(\hat{R}_{\textrm{new}},\epsilon ,a), \end{aligned}$$

as a function of the reproduction rate $\hat{R}_{\textrm{new}}$ of the new variant, the cross-immunity between invading and established variant $\epsilon$ and the immunity *a* against the established variant. For these simulations, we fixed the reproduction rate $\hat{R}_{\textrm{established}}$ of the established variant to 1.1. and evaluated our model on a grid varying $\hat{R}_{\textrm{new}}$, $\epsilon$ and *a* to evaluate resulting $\Delta t$. $\hat{R}_{\textrm{new}}$ was varied between 1.2 and 2.2 in 31 increments of 0.34, whereas $\epsilon$ and *a* were given each four different values as detailed above, yielding a total of 512 simulation scenarios.

## Results

### Regional fits and relative fitness of omicron against delta in metropolitan France

For metropolitan France in its entirety we find that the relative fitness of the omicron variant over the delta variant $\frac{\hat{R}_{\textrm{omicron}}}{\hat{R}_{\textrm{delta}}}$ is equal to 1.72. This value was obtained by assuming that 80% of the population was immunized against delta either by natural infection or vaccination and hence set $a=0.8$. Further we estimated that one fourth of this delta-immune population procured a partial immunity against omicron and thus set $\epsilon _{\mathrm {[delta,omicron]}} = 0.25$. Finally generation times of 5 days for the delta and 3.5 days for omicron variant where assumed respectively.

**Fig. 2:**
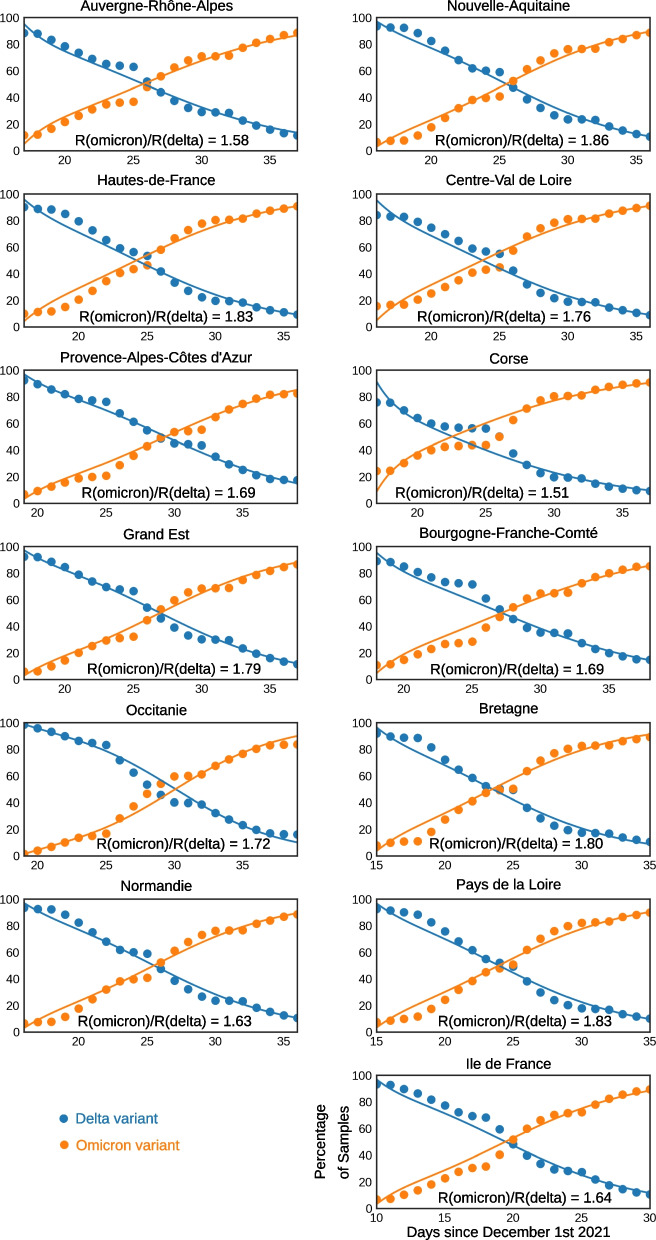
Omicron and delta SARS-CoV-2 variant proportions among positive samples in the regions respective window of opportunity, 10 days before and after the omicron variant exceeds 50%. Dots are representing proportions reported from sampled data published by *Santé Publique France*. Lines represent simulated data with estimated parameter values, here with a delta-omicron cross-immunity of 25% and an initial population that is 80% immunized against the delta variant. The generation time assumed here is 5 days for the delta variant and 3.5 days for the omicron variant

**Fig. 3:**
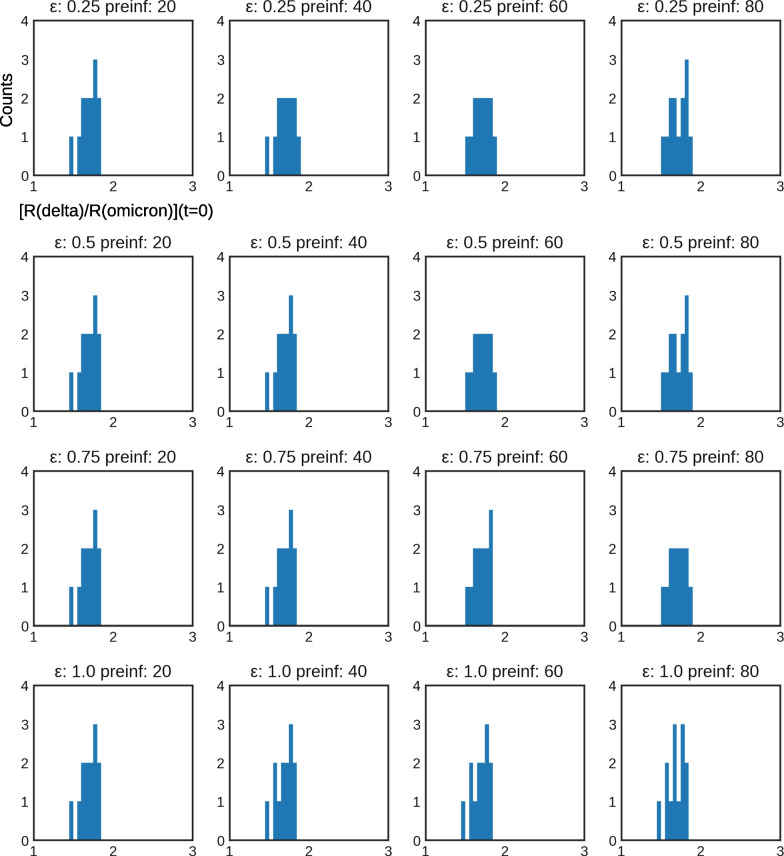
Distribution of $\frac{\hat{R}_{\textrm{omicron}}}{\hat{R}_{\textrm{delta}}}$ by regions shown by a 40 bins histogram between the values 1 and 3, when varying the cross-immunity $\epsilon$ and preimmunization level *a* (preinf). The generation time was 5 days for the delta variant and 3.5 days for the omicron variant

For this specific scenario, region specific assumed values for $\hat{R}_{\textrm{delta}}$ ans estimated values for $\hat{R}_{\textrm{omicron}}$ are displayed in Additional file 1: Table S1, and replacement curves and associated fits for all regions are reported in Fig. 2. The general trend is globally well reproduced by the model in all regions. The lowest fitness estimate was obtained for Corsica, with $\frac{\hat{R}_{\textrm{omicron}}}{\hat{R}_{\textrm{delta}}}=1.51$, while the highest for Nouvelle-Aquitaine, with $\frac{\hat{R}_{\textrm{omicron}}}{\hat{R}_{\textrm{delta}}}=1.86$. Distribution of fitness values is summarised in Fig. 3. The different regions assessed by this study feature variations in population density. Especially the Île-de-France region stands out with a population density larger than 1000 inhabitants per square kilometer. We investigated this matter as shown in Additional file 1: Fig. S9 in the supporting information and did not find any correlation between population density of a region and the relative transmission rates obtained for it.

### Sensitivity to uncertain model parameters

As a result of the robustness analysis on fitting, when the generation time of omicron is sampled at 3, 3.5, 4 and 6 days, we can remark that the relative strength of the omicron variant increases as the generation time approaches the generation time of the delta variant. The resulting shift in relative fitness can be observed in comparing Fig. 3 and Additional file 1: Fig. S1 to S3 or S4 to S7 if the data is interpreted with the alternative definition of omicron, found in the supporting information.

During our parameter sweep, the minimal relative fitness $\frac{\hat{R}_{\textrm{omicron}}}{\hat{R}_{\textrm{delta}}}$ of 1.38 is observed for Corsica as the generation time for omicron is set to 3 days. The Nouvelle-Aquitaine region exhibits the maximum value of 1.99 as the generation time for omicron is 4 days. Assuming a generation time of 5 days for delta and 3 days for the omicron variant we find that the average relative fitness $\frac{\hat{R}_{\textrm{omicron}}}{\hat{R}_{\textrm{delta}}}$, weighted by regional population size, ranged from 1.58 to 1.61. Increasing the generation time of omicron from 3 to 6, the relative fitness is increased to 2.31–2.34 in average. Estimates according to generation time assumptions are detailed in Table 2.

Estimates of omicron relative fitness do not seem to depend on the assumptions on cross-immunity conferred by previous immunity to delta in preventing omicron acquisition (Table 2 and Fig. 3). Varying the preimmunization levels of the population at the onset of the omicron invasion does not affect relative fitness values obtained from our model either. When varying these parameters, resulting fits and errors are similar to the ones shown in Fig. 2. The detailed outputs at region level have been made available on the Github repository^4^.

Note that, despite no effect of cross-immunity on omicron relative fitness was observed here, the corresponding transmission rate of the model $\beta$, as outlined in eq. (5), varies with variations of preimmunization *a* against delta and cross immunity $\epsilon$ by the predetermined factor $\frac{\gamma _{\textrm{omicron}}(1-a)}{\gamma _{\textrm{delta}}(1-a\epsilon _{\mathrm {[delta,omicron]}})}$, even if almost constant relative fitness rates $\frac{\hat{R}_{\textrm{omicron}}}{\hat{R}_{\textrm{delta}}}$ are reported in Table 2.

**Table 2:** Resulting estimates for a range of scenarios varying delta-to-variant cross-immunity ($\epsilon$), preimmunized populations proportion (*a*) and generation time (GT). The table displays $\hat{R}_{\textrm{omicron}}/\hat{R}_{\textrm{delta}}$ values (weighted mean across regions). Further minimum and maximum values for variance across regions as well as means of the loss function (6), both weighted by population size, are outlined at the bottom of each generation-time associated scenario .

	$a = 0.2$	$a = 0.4$	$a = 0.6$	$a = 0.8$
GT (delta 5, omicron 3) days				
$\epsilon$ = 0.25	1.589	1.591	1.595	1.607
$\epsilon$ = 0.5	1.592	1.565	1.594	1.608
$\epsilon$ = 0.75	1.590	1.590	1.593	1.603
$\epsilon$ = 1.0	1.588	1.587	1.585	1.580
Variance	$\sigma ^2 = [0.006-0.022]$			
Loss	$L = [1.26-1.85]$			
GT (delta 5, omicron 3.5) days				
$\epsilon$ = 0.25	1.705	1.707	1.711	1.724
$\epsilon$ = 0.5	1.704	1.706	1.711	1.725
$\epsilon$ = 0.75	1.705	1.705	1.707	1.720
$\epsilon$ = 1.0	1.704	1.704	1.702	1.696
Variance	$\sigma ^2 = [0.008-0.009]$			
Loss	$L = [1.39-1.42]$			
GT (delta 5, omicron 4) days				
$\epsilon$ = 0.25	1.823	1.824	1.827	1.838
$\epsilon$ = 0.5	1.823	1.823	1.826	1.838
$\epsilon$ = 0.75	1.823	1.823	1.824	1.835
$\epsilon$ = 1.0	1.823	1.822	1.820	1.814
Variance	$\sigma ^2 = [0.012-0.013]$			
Loss	$L = [1.48-1.54]$			
GT (delta 5, omicron 6) days				
$\epsilon$ = 0.25	2.317	2.321	2.328	2.344
$\epsilon$ = 0.5	2.317	2.319	2.325	2.343
$\epsilon$ = 0.75	2.317	2.324	2.340	2.340
$\epsilon$ = 1.0	2.316	2.315	2.312	2.307
Variance	$\sigma ^2 = [0.018-0.019]$			
Loss	$L = [1.65-1.73]$			

**Fig. 4:**
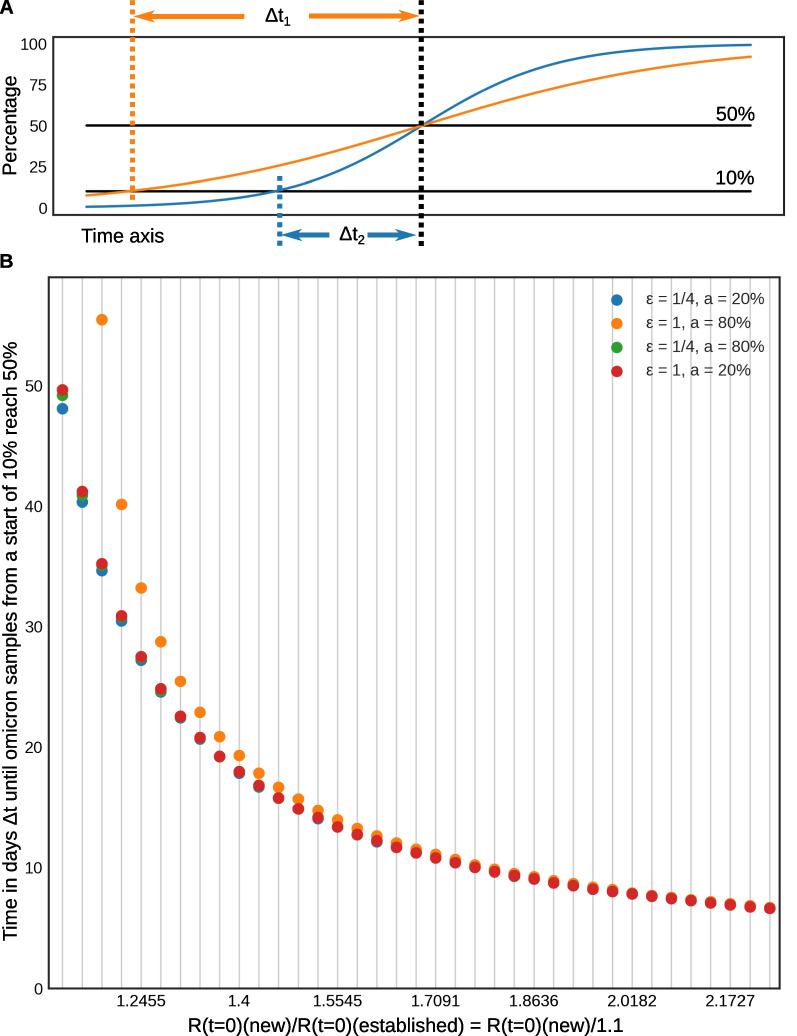
**A** Illustration for $\Delta t_{10\%-50\%}$ [defined in eq. (9)] taking two examples: a less fitter new variant (orange, $\Delta t_{1}$) and stronger new variant (blue, $\Delta t_{2}$) curves, respectively. **B** $\Delta t_{10\%-50\%}$ for different relative fitness $\hat{R}(t=0)_{\textrm{omicron}}/\hat{R}(t=0)_{\textrm{delta}}$, preimmunization (*a*) and cross-immunity ($\epsilon$) values. We see that in the regime of $\hat{R}(t=0)_{\textrm{omicron}}/\hat{R}(t=0)_{\textrm{delta}}> 1.5$, preimmunization and cross-immunity play a minor role in the steepness of variant replacement. On x-axis labels, “new” ans “established” stand for omicron and delta, respectively

Figure 4B outlines the expected values of $\Delta t$ as a function of the variant parameters as defined in eq. (9). Simulations show that, at relatively high fitness levels, as those found in real omicron and delta variant data $\left( \frac{\hat{R}_{\textrm{new}}}{\hat{R}_{\textrm{established}}} > 1.5\right)$, immunity and cross-immunity play a negligible role in the time needed, $\Delta t$, to reach the replacement of an established variant by a new fitter variant. In simulations, as $\frac{\hat{R}_{\textrm{new}}}{\hat{R}_{\textrm{established}}}$ rises, variations in preimmunization *a* and cross-immunity $\epsilon$ values become irrelevant and do not influence the time needed for a new variant to supplant an established one.

## Discussion

In this article we present a mechanistic model to simulate a multivariant epidemic of SARS-CoV-2. The model was used to analyse SARS-CoV-2 variant data reported by *Santé Publique France* between 1st of December 2021 and 30th of January 2022 on the proportions of different mutations occurring in a specially sampled subset of PCR tests at the French regional level. We detail how this model can be applied in order to evaluate the fitness of an emerging variant, relatively to established SARS-CoV-2 variants. Knowing the relative fitness of a new emerging variant against a previously observed variant, and having a model at hand to describe the dynamics of all variants is important for decision-makers in order to evaluate the risks caused by the epidemic and carefully plan future stress exhorted on public health systems, the economy and other affected areas.

Using the modeling framework outlined herein, we quantify the transmissibility advantage of the omicron variant as outlined in Table 2 and Figs. 2 and 3. The relative fitness between the omicron and delta variant is expressed by the fraction $\frac{\hat{R}_{\textrm{omicron}}}{\hat{R}_{\textrm{delta}}}$ and was found to range between 1.51 and 1.86 across regions when assuming that 80% of the population was immunized against delta, a cross delta_omicron cross-immunity of 25% and an omicron generation time parameterized to 3.5 days.

Figure 3 displays the increased transmissibility of the omicron variant compared to the delta variant as a distribution across all French regions. Using parameter sweeps, notably across different delta-omicron cross-immunities and preimmunity levels to the delta variant, we showed that this result was very robust and is almost not influenced by different hypotheses about the values of these two features at the beginning of December. These results are outlined in Table 2. Across all scenarios, our weighted average estimates of the increased transmissibility found for the omicron variant compared to the delta variant ranged from $\frac{\hat{R}_{\textrm{omicron}}}{\hat{R}_{\textrm{delta}}} = 1.57$ to $\frac{\hat{R}_{\textrm{omicron}}}{\hat{R}_{\textrm{delta}}} = 2.34$ over metropolitan French regions. It is worth mentioning that these results were obtained based on the absence of the L452R mutation only to identify omicron. Results for different variant indicators to describe omicron are highlighted in Additional file 1: Table S2 and Figures S3–S6.

Two studies provided early assessment of omicron initial spread in France [12, 13]. The study in [13] quantified the doubling time of omicron at the national level and for the Île de France and Centre-Val de Loire regions—the only two regions reporting substantial spread in the community at the time of the study—reporting values ranging between 1.8 and 2.5 days. The additive advantage in transmission was estimated at 105% by [12] from nationwide data. Our analysis considers the whole period of omicron replacement in all 13 regions in metropolitan France. Results confirm the rapid spread of omicron and provide estimates compatible with [12]. We highlight a variation in omicron fitness by region with a 20% deviation between the minimum and maximum transmission advantage relative to the weighted average across regions.

Our analysis is also comparable with previous studies made outside of France. Similar estimates of main parameters were indeed provided for Great Britain [23]. The British study finds an implied transmission advantage of omicron in the range of 160–210%. Prior studies from South Africa data find values either slightly above ours $\frac{\hat{R}_{\textrm{omicron}}}{\hat{R}_{\textrm{delta}}}$ [7] ranging from 1.8 to 3.2, with a mean variation over the month of November 2021 to be between 2.3 and 2.6, or in a second study [8] below our results with values for $\frac{\hat{R}_{\textrm{omicron}}}{\hat{R}_{\textrm{delta}}}$ lying between 0.75 and 2.0. Interestingly, a study using Danish data [9] estimated much higher values $\frac{\hat{R}_{\textrm{omicron}}}{\hat{R}_{\textrm{delta}}}$ 3.19 with a 95% confidence interval ranging from 2.82 to 3.61. Differences in the estimates may depend on different factors, including the surveillance protocols, the precise definition of the transmission advantage, and the modelling approach used for the data analysis.

The preimmunization levels of the population as well as different hypotheses about delta-omicron cross-immunity have almost no effect on the relative fitness estimates (Table 2 and Additional file 1: Table S2), possibly due also to identifiablity issues. Our simulations supported this result, showing that variations in $\epsilon _{\mathrm {[delta,omicron]}}$ and *a* are not expected to yield significant deviations in the dynamics of variant replacement when $\frac{\hat{R}_{\textrm{omicron}}}{\hat{R}_{\textrm{delta}}}$ is high enough ($> 1.5$) (Fig. 4).

The relative fitness $\frac{\hat{R}_{\textrm{omicron}}}{\hat{R}_{\textrm{delta}}}$ is however sensitive to hypotheses regarding differences in generation time between the delta and omicron variants. We investigated four values 3, 3.5, 4 and 6 days, for the generation time duration of the omicron variant, while keeping the delta variant at a constant generation time of 5 days. Although estimates distributions across regions were not changed, a shift towards higher values of $\frac{\hat{R}_{\textrm{omicron}}}{\hat{R}_{\textrm{delta}}}$ was observed as the generation time for the omicron was variant increased. Results for comparing different generation times are outlined in Table 2 and in Fig. 3 as well as in Additional file 1: Figs. S1-S5 in the supporting information. This result is coherent with a previously published analysis of omicron invasion in Great Britain [23].

During 2021, prior to the omicron wave, several studies have hypothesized different scenarios for the winter period 2021/2022. Sah et al. [27] have built several models to predict the situation in the USA. These authors state that immune escape has to be coupled with increased transmission rates for a variant to be successful. Dyson et al. [28] also explored, through various models, the dynamics of several hypothetical variants. The authors of [28] show that immune escape can slowly develop future waves that might not be easily predictable and can hit the population at later stages of the pandemic. Compared to our study, [27, 28] make long term predictions while we focus on the short period of quick replacement by omicron of the delta variant which lasted, as outlined, only 3 weeks.

As the analyzed data is not derived from whole genome sequencing but rather from the identification of specific mutations of the virus genome, the data published by *Santé Publique France* that tracks omicron strains allowed for different interpretations of the dataset. Here, we adopted the L452R mutation as an indicator for the delta variant, in presence of the mutation, and the omicron variant, in its absence. An alternative approach consists in combining the mutation L452R taken as a proxy for the delta variant with the set of omicron-specific mutations—deletion of site 69/70 and/or substitutions K417N and/or S371L-S373P and/or Q493R—as an indicator for omicron. The use of the two different indicators yields two distinct time series for each studied regions. Using only L452R both as an indicator for delta, in the presence of the mutation, as well as for omicron, in the absence of the mutation, yields less fluctuations in the time series, as outlined by the comparison of Fig. 2 and Additional file 1: Fig. S6. Using only this single mutation may in principle overestimate the omicron proportion at the onset of the omicron invasion, as a small non-delta fraction was continuously present in the dataset. However, the bias is likely to be limited in that full genome sequencing data showed that omicron become rapidly dominant among other variants without the L452R mutation during the first weeks of December [12, 13]. The time series based on multi-mutation definition were also analysed and relative fitness values $\frac{\hat{R}_{\textrm{omicron}}}{\hat{R}_{\textrm{delta}}}$ were evaluated. A larger omicron fitness was estimated as we analysed this second time series. This could be explained by the progressive mounting of this surveillance protocol concomitant with the omicron invasion, which may have biased the observation of the omicron dynamics. The different results for the two representations are outlined in the Table 2 and Additional file 1: Table S2. However, both definitions led to estimates that were consistent with those reported by various studies around the globe [7–9, 23].

No significant correlation between population density and effective omicron relative fitness as outlined by Additional file 1: Fig. S9 in the supporting information was observed. Several regional socio-demographic influencing factors could lead to changes in the spread of the different variants as outlined for instance in [29]. Focusing here on the relative fitness between omicron and delta, we did not pursue these issues further, as we found that population density appears to be decorrelated from the relative fitness between the two variants, and that the relative fitness does not seem to vary as strongly as demography.

## Conclusion

We estimated that as omicron replaced delta in France during winter 2021/2022, the relative fitness of the omicron variant compared to the delta variant, $\frac{\hat{R}_{\textrm{omicron}}}{\hat{R}_{\textrm{delta}}}$, ranges from 1.57 to 2.34. We propose here a multi-variant framework that enables short-term analysis of the epidemiological characteristics of an emerging variant using epidemiological data. The model presented here could be easily applied to other dynamic systems describing the evolution of epidemic diseases evolving into different variants.

## Supplementary Information

**Additional file 1.** Additional results of sensitivity analysis.

## Data Availability

The epidemiological model used to perform the analyses herein is available at https://github.com/haschka/SIER_multivariant_epidemic. Raw regional results are made available at https://github.com/haschka/French-Regional-Omicron-Invasion. Input data concerning regional variant sampling that has been processed herein is provided by *Santé Publique France* and available at: https://www.data.gouv.fr/fr/datasets/donnees-de-laboratoires-pour-le-depistage-indicateurs-sur-les-mutations/

## Footnotes

1 https://www.data.gouv.fr/fr/datasets/donnees-de-laboratoires-pour-le-depistage-indicateurs-sur-les-mutations/

2 https://github.com/haschka/SIER_multivariant_epidemic/

3 https://www.santepubliquefrance.fr/dossiers/coronavirus-covid-19/coronavirus-chiffres-cles-et-evolution-de-la-covid-19-en-france-et-dans-le-monde

4 https://github.com/haschka/French-Regional-Omicron-Invasion

## References

[1] Ge Y, Zhang W-B, Liu H, Ruktanonchai CW, Hu M, Wu X, Song Y, Ruktanonchai NW, Yan W, Cleary E, Feng L, Li Z, Yang W, Liu M, Tatem AJ, Wang J-F, Lai S (2022). Impacts of worldwide individual non-pharmaceutical interventions on COVID-19 transmission across waves and space. Int J Appl Earth Obs Geoinf.

[2] Viana R, Moyo S, Amoako DG, Tegally H, Scheepers C, Althaus CL, Anyaneji UJ, Bester PA, Boni MF, Chand M, Choga WT, Colquhoun R, Davids M, Deforche K, Doolabh D, Engelbrecht S, Everatt J, Giandhari J, Giovanetti M, Hardie D, Hill V, Hsiao N-Y, Iranzadeh A, Ismail A, Joseph C, Joseph R, Koopile L, Pond SLK, Kraemer MU, Kuate-Lere L, Laguda-Akingba O, Lesetedi-Mafoko O, Lessells RJ, Lockman S, Lucaci AG, Maharaj A, Mahlangu B, Maponga T, Mahlakwane K, Makatini Z, Marais G, Maruapula D, Masupu K, Matshaba M, Mayaphi S, Mbhele N, Mbulawa MB, Mendes A, Mlisana K, Mnguni A, Mohale T, Moir M, Moruisi K, Mosepele M, Motsatsi G, Motswaledi MS, Mphoyakgosi T, Msomi N, Mwangi PN, Naidoo Y, Ntuli N, Nyaga M, Olubayo L, Pillay S, Radibe B, Ramphal Y, Ramphal U, San JE, Scott L, Shapiro R, Singh L, Smith-Lawrence P, Stevens W, Strydom A, Subramoney K, Tebeila N, Tshiabuila D, Tsui J, van Wyk S, Weaver S, Wibmer CK, Wilkinson E, Wolter N, Zarebski AE, Zuze B, Goedhals D, Preiser W, Treurnicht F, Venter M, Williamson C, Pybus OG, Bhiman J, Glass A, Martin DP, Rambaut A, Gaseitsiwe S, von Gottberg A, de Oliveira T (2021). Rapid epidemic expansion of the sars-cov-2 omicron variant in Southern Africa. medRxiv.

[3] Thakur V, Ratho RK (2021). Omicron (b.1.1.529): a new sars-cov-2 variant of concern mounting worldwide fear. J Med Virol.

[4] Organization WH, et al. COVID-19 weekly epidemiological update, edition 70, 2021;2021.

[5] Mannar D, Saville JW, Zhu X, Srivastava SS, Berezuk AM, Tuttle KS, Marquez C, Sekirov I, Subramaniam S (2021). Sars-cov-2 omicron variant: Ace2 binding, cryo-em structure of spike protein-ace2 complex and antibody evasion. bioRxiv.

[6] Hu J, Peng P, Cao X, Wu K, Chen J, Wang K, Tang N, Huang A-l. Increased immune escape of the new sars-cov-2 variant of concern omicron. Cell Mol Immunol. 2022;19(2):293–5. 10.1038/s41423-021-00836-z. 10.1038/s41423-021-00836-z PMC8749347 35017716

[7] Abbott S, Sherratt K, Gerstung M, Funk S (2022). Estimation of the test to test distribution as a proxy for generation interval distribution for the omicron variant in England. medRxiv.

[8] Nishiura H, Ito K, Anzai A, Kobayashi T, Piantham C, Rodríguez-Morales AJ (2022). Relative reproduction number of sars-cov-2 omicron (b.1.1.529) compared with delta variant in South Africa. J Clin Med.

[9] Ito K, Piantham C, Nishiura H (2022). Relative instantaneous reproduction number of omicron sars-cov-2 variant with respect to the delta variant in Denmark. J Med Virol.

[10] Lundberg AL, Lorenzo-Redondo R, Ozer EA, Hawkins CA, Hultquist JF, Welch SB, Prasad PV, Oehmke JF, Achenbach CJ, Murphy RL, White JI, Havey RJ, Post LA (2022). Has omicron changed the evolution of the pandemic?. JMIR Public Health Surveill.

[11] France SP. COVID-19: Point épidémiologique hebdomadaire du 2021. Santé Publique France 2021.

[12] Sofonea MT, Roquebert B, Foulongne V, Verdurme L, Trombert-Paolantoni S, Roussel M, Haim-Boukobza S, Alizon S (2022). From delta to omicron: analysing the sars-cov-2 epidemic in France using variant-specific screening tests (September 1 to December 18, 2021). medRxiv.

[13] Mazzoli M, Di Domenico L, EMERGEN-Consortium, Colizza V. Early assessment of the omicron variant’s presence and growth rate in regions of France. Epix-lab reports 2021;(35).

[14] Andronico, Alessio and Tran Kiem, Cécile and Bosetti, Paolo and Paireau, Juliette: Impact du variant omicron sur l’épidémie covid-19 et son contrôle en france métropolitaine durant l’hiver 2021–2022. 2021.

[15] France SP. COVID-19: Point épidémiologique hebdomadaire du 2022. Santé Publique France 2022.

[16] Gangavarapu K, Latif AA, Mullen JL, Alkuzweny M, Hufbauer E, Tsueng G, Haag E, Zeller M, Aceves CM, Zaiets K, Cano M, Zhou J, Qian Z, Sattler R, Matteson NL, Levy JI, Lee RT, Freitas L, Maurer-Stroh S, Suchard MA, Wu C, Su AI, Andersen KG, Hughes LD (2022). Outbreak.info genomic reports: scalable and dynamic surveillance of sars-cov-2 variants and mutations. medRxiv.

[17] Gangavarapu K, Latif AA, Mullen JL, Alkuzweny M, Hufbauer E, Tsueng G, Haag E, Zeller M, Aceves CM, Zaiets K, Cano M, Zhou J, Qian Z, Sattler R, Matteson NL, Levy JI, Lee RT, Freitas L, Maurer-Stroh S, Suchard MA, Wu C, Su AI, Andersen KG, Hughes LD. French Variant Report: outbreak.info accessed 19 September 2022. https://outbreak.info/location-reports?loc=FRA. 2022.

[18] Khare, S., Gurry, C., Freitas, L., Schultz, M.B., Bach, G., Diallo, A., Akite, N., Ho, J., Lee, R.T., Yeo, W., Curation Team, G.C., Maurer-Stroh, S. (2021). GISAID’s role in pandemic response. China CDC Wkly.

[19] Sofonea MT, Roquebert B, Foulongne V, Morquin D, Verdurme L, Trombert-Paolantoni S, Roussel M, Bonetti J-C, Zerah J, Haim-Boukobza S, Alizon S (2022). Analyzing and modeling the spread of SARS-CoV-2 omicron lineages BA1 and BA2, France, September 2021-February 2022. Emerg Infect Dis.

[20] Mazzoli M, Di Domenico L, EMERGEN-Consortium, Colizza V. Assessment of the omicron ba.2 sub-lineage presence and growth rate in regions of France. Epix-lab reports. 2022;36.

[21] Gog JR, Grenfell BT (2002). Dynamics and selection of many-strain pathogens. Proc Natl Acad Sci.

[22] France SP. Communiqué de presse: Variant omicron: quelle surveillance mise en place? 2021.

[23] Abbott S, Sherratt K, Gerstung M, Funk S (2022). Estimation of the test to test distribution as a proxy for generation interval distribution for the omicron variant in England. medRxiv.

[24] Jones TC, Biele G, Mühlemann B, Veith T, Schneider J, Beheim-Schwarzbach J, Bleicker T, Tesch J, Schmidt ML, Sander LE, Kurth F, Menzel P, Schwarzer R, Zuchowski M, Hofmann J, Krumbholz A, Stein A, Edelmann A, Corman VM, Drosten C (2021). Estimating infectiousness throughout sars-cov-2 infection course. Science.

[25] Pagel C, Yates CA (2021). Tackling the pandemic with (biased) data. Science.

[26] Fehlberg E (1970). Klassische runge-kutta-formeln vierter und niedrigerer ordnung mit schrittweiten-kontrolle und ihre anwendung auf wärmeleitungsprobleme. Computing.

[27] Sah P, Vilches TN, Shoukat A, Fitzpatrick MC, Pandey A, Singer BH, Moghadas SM, Galvani AP (2021). Quantifying the potential dominance of immune-evading sars-cov-2 variants in the united states. medRxiv.

[28] Dyson L, Hill EM, Moore S, Curran-Sebastian J, Tildesley MJ, Lythgoe KA, House T, Pellis L, Keeling MJ (2021). Possible future waves of sars-cov-2 infection generated by variants of concern with a range of characteristics. Nat Commun.

[29] Mogi R, Spijker J (2021). The influence of social and economic ties to the spread of COVID-19 in Europe. J Popul Res.

